# Shift in therapeutic approaches in patients with hypercholesterolemia - a secondary data analysis

**DOI:** 10.1186/s12875-025-02982-z

**Published:** 2025-09-25

**Authors:** Elena Zink, Jost Steinhäuser, Paul-Georg Blickle, Wolfgang C. G. von Meißner, Christoph Strumann

**Affiliations:** 1 Institute of Family Medicine, University Medical Centre Schleswig-Holstein, Ratzeburger Allee 160, Lübeck, 23538 Germany; 2 Hausärzte am Spritzenhaus, Baiersbronn, Germany; 3 CBS - University of Applied Sciences, Köln, Germany

**Keywords:** Hypercholesterolemia, Guideline, Primary care, Statin, Cardiovascular diseases

## Abstract

**Background:**

Atherosclerotic cardiovascular disease is a leading cause of death and hypercholesterolemia is one relevant risk factor. There are two established guidelines for the management of high cholesterol in general practice in Germany, one of these is from the European Society of Cardiology (ESC). This analysis examines whether treatment modalities and clinical outcomes in German primary care have changed after the publication of the 2019 ESC (European Society of Cardiology)/EAS (European Atherosclerosis Society) guidelines on dyslipidaemia.

**Methods:**

A retrospective cohort study (2001–2023) with data extracted from electronic health record systems of 17 primary care practices. The Data was used to compare drug treatment and outcomes before and after 2019, the year of the ESC guideline publication. Multilevel regression analysis was used to assess the effects of the new guidelines, accounting for time trends and patient- and practice-level factors.

**Results:**

Statin prescriptions for 23,322 patients with hypercholesterolemia increased from 6% in 2001 to 27% in 2023, with an increase in treatment intensity after 2012. Despite general reductions in total and LDL cholesterol, cardiovascular events remained unchanged, while reported complaints associated with side effects increased. Multilevel regression showed more prescriptions after the new ESC/EAS guidelines, with no effect on treatment intensity or outcomes.

**Conclusion:**

Following the publication of the new ESC/EAS guideline, a modest rise in statin prescriptions has been documented. However, despite improved lipid profiles, clinically relevant events did not decrease yet, and side effects increased, questioning the benefit of tailoring statin therapy to lower lipid thresholds in real world data so far.

**Trial registration:**

German Clinical Trials Register: DRKS00032936. Registration Date: October 30, 2023.

**Supplementary Information:**

The online version contains supplementary material available at 10.1186/s12875-025-02982-z.

## Background

Ischemic heart disease (IHD), which is typically caused by obstructive coronary atherosclerosis, is recognised as the leading cause of cardiovascular disease (CVD)-related deaths, with stroke being the second most common cause. In European Society of Cardiology (ESC) member countries, IHD accounts for 45% of all CVD-related deaths in women and 39% in men [1]. Certain behaviours, such as consuming an unhealthy diet, smoking, and being physically inactive can lead to high blood pressure, dyslipidaemia, diabetes and obesity, which increase the risk of developing an ischemic heart disease or stroke [2]. Therefore, for prevention it is essential that healthcare professionals identify patients’ individual risk factors, as these can be modified to some extent.

One of the risk factors for which the effects of modification have been studied extensively is hypercholesterolemia [3]. About 39% of adults worldwide have levels of total cholesterol that are labelled as elevated [4]. In accordance with the level of cardiovascular risk, the intervention should always include lifestyle modifications, e.g. regular physical activity or quitting to smoke [3, 5, 6].

Pharmacological interventions are suggested if reducing the cholesterol level is insufficient [5]. Studies suggest a reduction of around 20% in the relative risk of major cardiovascular events for every 1 mmol/L of LDL cholesterol (LDL-C) lowering achieved with statin therapy [7, 8].

In Germany, two different guidelines exist for treating hypercholesterolemia in general practice. The one of the German College of General Practitioners and Family Physicians (DEGAM) recommends drug therapy with statins in a fix dose in cases of elevated cholesterol levels and increased cardiovascular risk [9]. Whereas, the one of the European Society of Cardiology (ESC) and European Atherosclerosis Society (EAS) recommends dosing statins according to LDL-C target ranges varying by risk profile. An updated version of this ESC/EAS guideline released in 2019 includes a recommendation for lower levels than in the previous edition [5].

Regarding the integration into clinical practice, the objective of this study was to consider whether and to what extent the revised ESC/EAS recommendations on lipid management in patients in a primary prevention situation have resulted in a shift in prescriptions and therapeutic approaches in primary care in Germany.

## Methods

### Study design and data collection

We conducted a retrospective cohort study with routine data (from 2001 to 2023) from 17 primary care practices of the Supraregional Health Service Research Network (SHRN), Germany to compare drug treatment and treatment outcomes before and after 2019. The sample included all patients (over 300,000 different patients), who at least have been to one of the primary care practices. Relevant routine data from the electronic health record systems (including sociodemographic information, data about the practice visits (diagnoses and prescriptions), laboratory test results, as well as permanent diagnoses that patients have received prior to the sample period) were extracted for the analysis. Patients showing up for pandemic reasons only (testing or getting vaccinated) were excluded. Details about the data processing can be found in Strumann et al. [10].

Patients with the diagnosis hyperlipidaemia (ICD-10 Code E78) were identified based on information stored in the data. The analysis is focused on the primary prevention of patients with hypercholesterolemia, excluding those who have experienced a myocardial infarction or stroke.

### Study variables

This analysis examined three different aspects of the consequences of a potential change in lipid management.

First, we examined the possibility of an increase in the number of patients who were receiving statin prescriptions as well as those who were either increasing the dosage of their statin therapy or whose therapy had been escalated. In this context, therapy escalation refers to switching to a more effective statin or combining it with another lipid-lowering agent. The latter two measures were calculated as cumulative proportions, i.e., in the event of an escalation or dose increase, the patient will continue to be identified as such in subsequent years unless the escalation or dose is reduced. Considering the proposed guideline recommending lower treatment targets for statin therapy, it is expected that the proportion of patients in all three previously mentioned groups will increase following the year 2019.

A secondary objective was to ascertain how the introduction of the new guideline affected the health status of patients. The lipid profile of the patients (LDL-C and total cholesterol) was examined, as well as the clinically relevant events that occurred. These included the incidence of myocardial infarctions (ICD-10 I21, I22) and strokes (ICD-10 I63, I64). The expected effect of the recommended, more intense statin therapy is a reduction in both.

A third aspect was an examination of the side effects, which were classified as diagnoses or keywords identified from free-text search of specific text fields including the anamnesis and clinical results, representing the most common adverse effects of statin treatment in patients receiving statin therapy. These included muscle pain, liver dysfunction and dyspepsia/flatulence, as well as diagnoses categorised as adverse drug reactions [11] (for details see Table S1 in the supplements). The use of high-dose statins is expected to cause more side effects beyond 2019.

### Statistical analysis

The statistical analyses were conducted on an annual level for each considered patient. The effects of the publication of the new guideline were determined using a before-after comparison. Given that all patients were simultaneously exposed to potential alterations in the management strategy, in addition to a graphical inspection, a multivariate multilevel regression analysis was conducted. To minimize the risk of a biased estimated guideline effect due to potential confounding variables, we specified a general cubic time trend and controlled for time-invariant heterogeneity among the patients (e.g., individual susceptibility to disease/family history) and the practices (e.g., location, equipment) by specifying corresponding individual effects. In addition to gender, time-varying patient-level control variables from the previous year such as the number of diagnoses (ICD-Chapter) per practice visit, the number of practice visits, hospitalizations, specialist visits and whether the patient was referred to a cardiologist as well as specific risk factors (smoking, hypertension, diabetes mellitus, asthma/chronic obstructive pulmonary disease, obesity and migraine/chronic headache) were also included.

## Results

### Sample characteristics

A total of 310,963 patients were treated in one of the 17 practices leading to 1,659,151 annual observations. Of these patients, 23,322 (7.5%) were diagnosed with hypercholesterolemia without the occurrence of a myocardial infarction or stroke (primary prevention) with 186,450 annual observations. In Table 1, characteristics of the patients and differences between patients with hypercholesterolemia and non-hypercholesterolemic patients are shown. All differences are significant at the 0.1% level, except for migraine/chronic headache.

**Table 1:** Sample characteristics, n (%)

Variable	all	hypercholesterolemic	non-hypercholesterolemic
patients	310,963	23,322	302,060
annual observations	*n* = 1,659,151	*n* = 186,450 (11.2%)	*n* = 1,472,701 (88.8%)
sociodemographic			
*female*	888,430(53.5)	98,691(52.9)	789,739(53.6)
*male*	766,964(46.2)	87,706(47.0)	679,258(46.1)
*age*,* µ(SD)*	45.2(21.8)	62.4(14.7)	43.0(21.6)
health service demand			
*number of diagnoses at practice visit (ICD‑Chapter)*,* µ(SD)*	1.0(1.7)	2.7(2.7)	0.8(1.5)
*number of practice visits*,* µ(SD)*	4.1(6.5)	7.9(7.8)	3.7(6.1)
*number of hospitalizations*,* µ(SD)*	0.3(1.3)	0.6(1.9)	0.3(1.2)
*hospitalization (yes)*	199,400(12.0)	41,105(22.0)	158,295(10.7)
*number of specialist-visits*,* µ(SD)*	1.9(4.0)	4.2(5.3)	1.7(3.7)
*specialist-visit (yes)*	750,318(45.2)	135,355(72.6)	614,963(41.8)
*number of cardiologist-visits*,* µ(SD)*	0.1(0.7)	0.3(1.1)	0.1(0.6)
*cardiologist-visit (yes)*	81,461(4.9)	25,152(13.5)	56,309(3.8)
risk factors^a^			
*smoking*	41,817(2.5)	8,900(4.8)	32,917(2.2)
*hypertension*	160,033(9.6)	52,128(28.0)	107,905(7.3)
*diabetes mellitus*	68,691(4.1)	25,382(13.6)	43,309(2.9)
*asthma/chronic obstructive pulmonary disease*	101,262(6.1)	17,032(9.1)	84,230(5.7)
*obesity*	31,787(1.9)	9,882(5.3)	21,905(1.5)
*migraine/chronic headache*	33,152(2.0)	3,792(2.0)	29,360(2.0)
Cholesterol level (one year before/after the diagnosis (mg/dl)^b^)			
*total*,* µ/N(SD)*	209.9/26,094(47.8)	233.1/8,989(46.7)	197.6/17,105(43.7)
*LDL-C*,* µ/N(SD)*	134.2/19,000(40.8)	152.8/6,733(40.6)	123.9/12,267(37.1)

310,963 patients and 23,322 (7.7%) patients with hypercholesterolemia. µ: mean, N: number of annual observations. All differences between both groups are significant at the 0.1% level (p<0.001), except for migraine/chronic headache. ^a^Risk factors were measured when a respective diagnosis was documented in a given year for each patient. Smoking was also considered if it was documented outside of the diagnostic documentation in the electronic health record systems. ^b^For non-hypercholesterolemic patients, the most recent cholesterol level (before the diagnosis of hyperlipidaemia) was used. For hypercholesterolemic patients, the cholesterol level immediately after the respective diagnosis of hyperlipidaemia was used.

Regarding the sociodemographic variables, patients with hypercholesterolemia were, on average, 19.4 years older than non-hypercholesterolemic patients. The percentage of men was higher in the group of patients with hypercholesterolemia (47.0% vs. 46.1%). These patients have in general a higher demand for health services and higher prevalence of risk factors. Both cholesterol measures, total and LDL-C, were higher for the group of hypercholesterolemic patients (total cholesterol: 233.1 vs. 197.6).

### Development of medication and outcomes

**Fig. 1:**
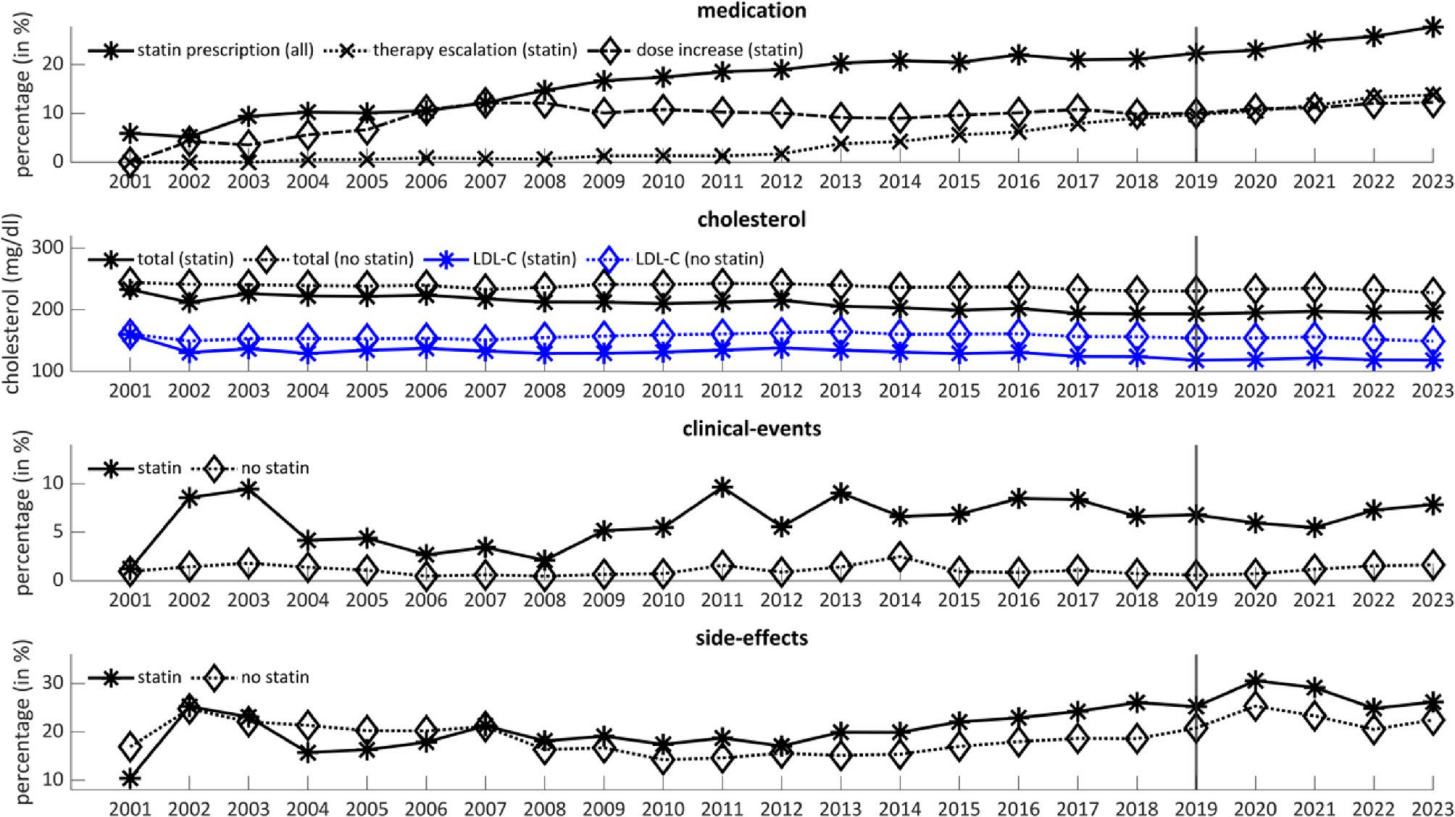
Medication and outcomes over time

Figure 1 shows the development of the therapeutic medication and outcomes of hypercholesterolemic patients over time from 2001 to 2023. The percentage of patients receiving statin prescriptions increased steadily over the total period, beginning at 6–27% in 2023. After 2012, the increase in therapeutic escalation accelerates up to 14% of all patients with a statin prescription and hypercholesterolemia. The percentage of patients receiving a higher dose has doubled from 2003 to 2007 and remained rather stable until 2023. For a comprehensive overview of the statins that have been prescribed, along with the respective doses, readers are referred to Tables S4 in the supplementary material. In Table S5, the distribution of lipid-lowering agents is provided, incorporating both non-statin agents and statins. As shown by Table S5, non-statin lipid-lowering therapies were prescribed for less than 0.5% of the patients, while statin lipid-lowering combinations were not prescribed at all.

The developments of the outcome variables are shown separately for patients prescribed and not prescribed statins. Patients who received a statins prescription had lower cholesterol levels than other patients. For both groups, cholesterol level declined steadily over time. The number of clinical events was higher in patients prescribed statins. The trend over time was rather stable for both groups, however, it exhibits a higher degree of variation for patients who have been prescribed statins. The percentage of patients with documented side effects was higher in the group of patients receiving statins. In both groups, there was an increase after 2014. Figure S1 in the supplements provides a more detailed overview of the proportion of each subcategory of the side effects.

### Multivariate analysis

Table 2 shows the estimated effects of the introduction of the revised ESC/EAS recommendations on lipid management in patients with a diagnosis of hypercholesteremia on prescriptions and therapeutic approaches as well as on patients’ outcomes. For the therapy escalation and dose increase, only patients were considered that received a statin prescription. For the outcome variables, the regression models control for the group of patients receiving a statin prescription and an interaction effect between this group and the introduction of the guideline is specified. A cubic trend specification was selected for all models.

**Table 2:** Multivariate analysis

Medication		statin prescription	therapy escalation	dose increase
Model		(1)	(2)	(3)
		Logistic	Logistic	Logistic
after 2019	OR^a^	1.13^*^	0.58	0.96
	*p*-value	(0.0345)	(0.0747)	(0.8556)
	95%CI	[1.01,1.26]	[0.32,1.06]	[0.65,1.44]
	N	159,044	32,514	32,514
Outcomes		total cholesterol	clinical events	side effects
Model		(4)	(5)	(6)
		Linear	Logistic	Logistic
statin prescription	$\:\widehat{\beta\:}$/OR^a^	−24.82***	13.46***	1.27***
	*p*-value	(< 0.0001)	(< 0.0001)	(< 0.0001)
	95%CI	[−25.8,−23.9]	[11.1,16.3]	[1.21,1.34]
after 2019	$\:\widehat{\beta\:}$/OR^a^	7.16***	0.79	1.41***
	*p*-value	(< 0.0001)	(0.1049)	(< 0.0001)
	95%CI	[5.74,8.57]	[0.57,1.01]	[1.30,1.52]
after 2019 $\:\times\:$statin prescription	$\:\widehat{\beta\:}$/OR^a^	−7.06***	1.51***	1.03
	*p*-value	(< 0.0001)	(0.0013)	(0.4488)
	95%CI	[−8.42,−5.70]	[1.17,1.93]	[0.95,1.11]
	N	52,146	159,044	159,044

^a^*OR* Odds Ratio,$\:\widehat{\beta\:}$: estimated coefficients; values of the control variables were measured in the previous year, their estimated coefficients/Odds Ratios are not shown (see Table S2 and S3 in the supplement for detailed presentation of the regression results); individual practice and patient level effects; *p*-values in parentheses; 95% confidence intervals in brackets; significance levels: ^*^p < 0.05, ^**^p < 0.01, ^***^p < 0.001

The results of the multilevel regression analysis suggest that there was a rather small increase of statin prescriptions after the revised ESC/EAS recommendations introduction; the odds of receiving a statin prescription were increased by a factor of 1.13 (95%CI: [1.01,1.26]). Such an effect cannot be observed for therapy escalation and dose increase. Regarding the outcome variables, the results indicated that patients who have been prescribed a statin medication exhibit lower total cholesterol levels of 24.82 mg/dL in comparison to the other patients. Furthermore, among patients receiving a statin prescription more clinical events (Odds Ratio: 13.46, 95%CI: [11.1,16.3]) as well as more side effects (Odds Ratio: 1.27, 95%CI: [1.21,1.34]) were observed. Total cholesterol levels and the likelihood of patients reporting complaints associated with side effects increased significantly after 2019. However, the latter effect was not significantly different between patients who received a statin prescription and those who did not (Odds Ratio: 1.03, 95%CI: [0.95,1.11]). Clinical events have not changed after the introduction of the new guidelines.

As a supplemental analysis, event-study plots for total cholesterol levels and side effects were generated using two-way fixed effects models with the same control variables as above (see Figure S2 in the supplement). The purpose of this analysis was to illustrate the temporal dynamics of the observed associations in relation to the initiation of statin therapy. For clinical events, no such plot could be created due to missing follow-up data after the event. The plots show a substantial and sustained decrease in total cholesterol levels following the first statin prescription, with no notable trends in the years prior. For side effects, a sharp increase is observed immediately after treatment initiation, while the estimated Odds-Ratios remain slightly elevated but insignificant in subsequent years, except for the years 10 and 11 after the first statin prescription.

## Discussion

In our analysis we examined, whether prescriptions and treatment approaches observed in primary care in Germany has changed after the introduction of the revised ESC/EAS recommendations on lipid management in patients with the cardiovascular risk factor of hypercholesterolaemia. As the results demonstrated, there was a steady increase in statin prescriptions over the last 23 years, however, not a substantial increase after the revised ESC/EAS guideline in 2019. Furthermore, there was no increase in dose or switch to more effective statins after 2019. The use of statins improved lipid profiles and increased side effects. However, no reduction in clinically relevant events was observed as a result of drug therapy yet.

As our analysis has shown, there has been a small but statistically significant increase in the number of statin prescriptions issued by general practitioners in the years after the introduction of the updated ESC/EAS guideline in 2019. A rise in statin prescriptions has also been seen in other countries in past years, for example in Croatia [12]. However, it is notable that a growing proportion of prescriptions for these drugs had already been observed in previous years. One potential explanation for this could be the introduction of numerous generic medications in recent years. For example, our analysis indicates that the year 2012 represents a significant point in time, possibly marking the introduction of generic atorvastatin to the German market [13]. Following this date, there is a notable surge in the prescription of more efficacious statins. Conversely, if there was a connection with the introduction of the updated guideline, one would expect that more statins in higher doses and more effective statin preparations were prescribed than in previous years [5, 14]. In contrast, an intensified lipid-lowering medication for secondary prevention was observed among patients with coronary artery disease participating in an ambulatory cardiac rehabilitation program in Switzerland [15].

Another recommendation of the guideline that is not reflected in our observations is the use of combination therapy with non-statin lipid-lowering agents, particularly when the target value cannot be achieved with statins alone [5]. Studies have shown that combined lipid-lowering therapy results in a greater reduction in LDL-C with the same risk of side effects and a lower risk of cardiovascular events [16]. Accordingly, a higher prescription frequency of non-statin lipid-lowering therapies (LLTs) would now be expected to be observed than in our analysis. A recent study shows that, in 2021, over 90% of therapies were statin monotherapies, with only a small proportion of non-statin LLTs being prescribed, either in combination with a statin or alone [17].

In our patient cohort, there has been no notable reduction in the incidence of myocardial infarction and stroke in recent years, although a larger proportion of the patients observed were prescribed a statin. This is an interesting finding as there are studies that demonstrate a correlation between the initial LDL cholesterol level and the efficacy of therapy in reducing the risk of a cardiovascular event [18] and others that detect LDL-C levels as a surrogate marker with only weak associations with clinical outcomes [19].

Similarly, although there has been a reduction in total cholesterol and LDL cholesterol in the patient population in recent years, a slight increase was observed after 2019, contrary to the expected trend. This can be influenced by several factors. For example, patient adherence represents an additional factor that was not explicitly considered in our analysis. Research has shown that non-adherence to therapy by patients is a major barrier to optimal treatment for patients [20]. In this context, research studies have indicated that a low level of adherence to statin therapy is associated with an elevated risk of mortality [21]. Additionally, other physician-related barriers to guideline adherence have been identified. Disagreement with the recommendations and the belief that the patient does not require treatment are frequently cited as reasons for non-adherence [22]. Moreover, different guidelines on the same topic may offer divergent recommendations. This is evident in the two previously mentioned guidelines for the treatment of hypercholesterolemia in Germany, for example [5, 9]. While the ESC/EAS guideline advocates an intensified strategy, the DEGAM guideline suggests a more conservative approach. Specifically, it recommends fixed-dose therapy without a target value and no combination therapy with non-statin LLTs [9]. Consequently, it may be that general practitioners in the observed practices tend to adhere more to the DEGAM guideline than the recently updated ESC guideline. This could explain why there has been no observed increase in all domains of statin therapy after 2019. Further research would be needed to address this question. In order to assess the influence of a guideline on the treatment of a specific patient population, it is also essential to consider the extent to which the guideline has been implemented. Existing literature on this subject, including the SANTORINI study [23], clearly demonstrates a significant delay in the implementation of guidelines in clinical practice. Even after a considerable time, the recommendations have not been implemented to a satisfactory standard [24]. This is a relevant factor that must be considered when interpreting the data.

The dissemination of a guideline update could help to positively influence hypercholesterolaemia. However, it appears that a clinically relevant effect regarding cardiovascular events is not achievable from a certain point onwards by further increasing the dose. In the context of the German health policy agenda, the topic of preventing Atherosclerotic Cardiovascular Disease (ASCVD) has been the subject of considerable discussion under the previous government until recently. The “Healthy Heart Act” was labelled as having the aim to strengthen the implementation of risk prevention measures for cardiovascular disease starting at young age with screening measures. The legislation would also have allowed the use of statins at a lower threshold and with greater frequency [25, 26]. Future studies should calculate the number needed to treat versus the number needed to harm in a situation where new standard values are proposed before such acts pass the legislation.

### Strengths and limitations

This is the first study to examine the management of dyslipidaemias using real-world data on a large scale, encompassing a substantial number of patients and data over two decades, drawn from primary care practices in Germany. The data used provide unique insights into the relationship between therapeutic management and patient outcomes in light of the implementation of a new guideline. Although we only used data from 17 primary care practices, our data offers additional benefit to those from a clinical trial, as these typically focuses on a homogeneous patient population due to its narrow selection criteria. In our data, 7.5% of all patients had a diagnosis of hypercholesterolemia. This is quite in line with findings from the CONTENT project, a source of prevalence in primary care in Germany, the overall prevalence of hypercholesterolemia was there reported to be 8.5% [27].

Despite these advantages, the use of real-world data has also some inherent weaknesses, most notably low internal validity. This applies especially to the operationalization of the side effects. We cannot guarantee whether the reported complaints were associated to the statin prescription, producing a lot of statistical “noise”. Assuming that these noise patterns are unsystematic, the results of the multilevel regression analysis should be robust to the low internal validity of the side effect variable. Moreover, the event-study plot shows a clear and immediate increase in reported side effects following statin initiation, suggesting that—despite the low internal validity—the variable captures treatment-related variation. Consequently, the detected associations are not solely attributable to unsystematic noise. While the effects on treatment escalation and cholesterol as well as on side effects can be considered as short-term, the clinical events considered (i.e., stroke and myocardial infarction) may have some latency in the effect. Further, there is also a time lag between the introduction of a guideline and its implementation [23]. To take this into account, a longer time horizon after the 2019 guideline implementation should consider in future studies. Further, the statistical methods used do not allow causal statements to be made about the relationship between medication and patient outcomes.

## Conclusion

In conclusion, the present study shows that a small increase in statin prescriptions was observed after the publication of the revised ESC/EAS guideline. However, despite the observed improvements in lipid levels, there was no reduction in clinically relevant events observable yet but an increase in adverse events.

## Supplementary Information

Supplementary Material 1.

## Data Availability

The data sets generated and analysed in the current study are not publicly available due to ethical or privacy reasons. However, the datasets used and analysed during the current study are available from the corresponding author on reasonable request with the permission from the individual practices of the SHRN.
