# Supplementary material for: Shift in therapeutic approaches in patients with hypercholesterolemia - a secondary data analysis
Source: BMC Prim Care. 2025 Sep 25;26:287. doi: 10.1186/s12875-025-02982-z (PMC12462300; doi:10.1186/s12875-025-02982-z)
Supplement: Supplementary file 1 — Supplementary Material 1. [file 12875_2025_2982_MOESM1_ESM.pdf]

Supplements

Table S1. ICD-10 and ICPC2 codes used to identify side-effects of statin prescription

ICD-10-Codes	ICPC2-Codes*
Muscular complaints	
M79.1 Myalgia	L18 Muscle Pain
G72.0 Drug-induced myopathy	L19 Muscle symptom/complaint NOS
G72.9 Myopathy, unspecified	L29 Symptom/complaint Musculoskeletal other
R74.8 Abnormal levels of other serum enzymes	
Dyspepsia, Flatulence	
R10.1 Pain localized to upper abdomen	D01 Abdominal pain/cramps general
R10.4 Other and unspecified abdominal pain	D02 Abdominal pain epigastric
R11 Nausea and vomiting	D07 Dyspepsia/indigestion
R12 Heartburn	D08 Flatulence/gas/belching
R14 Flatulence and related conditions	D09 Nausea
R19.4 Change in bowel habit	D18 Change faeces/bowel movements
K30 Functional dyspepsia	D25 Abdominal distension
K52.9 Noninfective gastroenteritis and colitis, unspecified	
K56.4 Other impaction of intestine	
K59.0 Constipation	
K59.1 Functional diarrhoea	
Liver dysfunction	
K71 Toxic liver disease	D97 Liver disease NOS
K72 Hepatic failure, not elsewhere classified	
K76 Other diseases of liver	
K77 Liver disorders in diseases classified elsewhere	
R10.1 Pain localized to upper abdomen	
R53 Malaise and fatigue	
R63.4 Abnormal weight loss	
R74.0 Elevation of levels of transaminase and lactic acid dehydrogenase [LDH]	
R74.8 Abnormal levels of other serum enzymes	
R94.5 Abnormal results of liver function studies	
Adverse drug reaction	
I95.2 Hypotension due to drugs	A85 Adverse effect medical agent
J70.2 Acute drug-induced interstitial lung disorders	
J70.3 Chronic drug-induced interstitial lung disorders	
J70.4 Drug-induced interstitial lung disorders, unspecified	
L27.0 Generalized skin eruption due to drugs and medicaments	
T88.7 Unspecified adverse effect of drug or medicament	
T88.8 Other specified complications of surgical and medical care, not elsewhere classified	
T88.9 Complication of surgical and medical care, unspecified	

*ICPC2: Classification of Primary Care-Version 2. Lamberts, H., & Wood, M. (2002). The birth of the International Classification of Primary Care (ICPC) Serendipity at the border of Lac Leman. Family Practice, 19(5), 433-435.

Table S2. Multivariate analysis: Medication

**Medication**		**statin prescription**	**therapy escalation**	**dose increase**
	Model	(1) Logistic	(2) Logistic	(3) Logistic
after 2019	OR	1.13*	0.58	0.96
	p-value	(0.0345)	(0.0747)	(0.8556)
	95%CI	[1.01,1.26]	[0.32,1.06]	[0.65,1.44]
t	OR	1.69***	5.16*	15.76***
	p-value	(<0.0001)	(0.0193)	(<0.0001)
	95%CI	[1.59,1.80]	[1.30,20.4]	[10.5,23.6]
t^2	OR	0.97***	0.98	0.84***
	p-value	(<0.0001)	(0.7296)	(<0.0001)
	95%CI	[0.97,0.98]	[0.90,1.08]	[0.82,0.87]
t^3	OR	1.00***	1.00	1.00***
	p-value	(<0.0001)	(0.5048)	(<0.0001)
	95%CI	[1.00,1.00]	[1.00,1.00]	[1.00,1.00]
female	OR	0.49***	0.82	0.43***
	p-value	(<0.0001)	(0.4974)	(<0.0001)
	95%CI	[0.43,0.55]	[0.46,1.45]	[0.30,0.64]
age (reference: >80)				
<50	OR	0.12***	0.14**	0.43*
	p-value	(<0.0001)	(0.0046)	(0.0176)
	95%CI	[0.11,0.14]	[0.04,0.55]	[0.22,0.86]
50-65	OR	0.40***	0.39**	0.34***
	p-value	(<0.0001)	(0.0087)	(<0.0001)
	95%CI	[0.36,0.45]	[0.19,0.79]	[0.22,0.53]
65-80	OR	1.00	0.82	0.95
	p-value	(0.9783)	(0.5023)	(0.7660)
	95%CI	[0.92,1.09]	[0.46,1.46]	[0.68,1.33]
health service demand of the previous year				
practice visits	OR	1.02***	1.04***	1.02*
	p-value	(<0.0001)	(0.0006)	(0.0485)
	95%CI	[1.02,1.03]	[1.02,1.07]	[1.00,1.03]
hospitalization	OR	0.95***	0.97	1.02
	p-value	(<0.0001)	(0.2327)	(0.4409)
	95%CI	[0.94,0.96]	[0.91,1.02]	[0.97,1.06]
specialist visit	OR	1.03***	1.03	1.04***
	p-value	(<0.0001)	(0.1344)	(0.0001)
	95%CI	[1.03,1.04]	[0.99,1.06]	[1.02,1.07]
cardiologist visit	OR	1.51***	1.76***	1.75***
	p-value	(<0.0001)	(<0.0001)	(<0.0001)
	95%CI	[1.42,1.61]	[1.24,2.50]	[1.39,2.21]
risk factors (measured in the previous year)				
smoking	OR	1.27***	1.43	1.30
	p-value	(<0.0001)	(0.1589)	(0.1405)
	95%CI	[1.15,1.41]	[0.87,2.34]	[0.92,1.85]
hypertension	OR	1.16***	1.15	0.92
	p-value	(<0.0001)	(0.4346)	(0.4563)

	95%CI	[1.09,1.23]	[0.81,1.65]	[0.73,1.16]
diabetes mellitus	OR	2.03***	1.42	1.25
	p-value	(<0.0001)	(0.1382)	(0.1252)
	95%CI	[1.87,2.21]	[0.89,2.27]	[0.94,1.66]
asthma/chronic obstructive pulmonary disease	OR	0.97	1.10	1.33
	p-value	(0.4544)	(0.7636)	(0.1048)
	95%CI	[0.89,1.05]	[0.61,1.98]	[0.94,1.87]
obesity	OR	0.81**	0.59	0.80
	p-value	(0.0010)	(0.2213)	(0.4092)
	95%CI	[0.71,0.92]	[0.26,1.37]	[0.47,1.37]
migraine/chronic headache	OR	0.80*	0.21*	0.58
	p-value	(0.0272)	(0.0208)	(0.2143)
	95%CI	[0.66,0.98]	[0.05,0.79]	[0.24,1.38]
	n	159044	32514	32514

OR: Odds Ratio; individual practice and patient level effects; *p*-values in parentheses; 95% confidence intervals in brackets; significance levels: $^{*}p < 0.05$, $^{**}p < 0.01$, $^{***}p < 0.001$

Table S3. Multivariate analysis: Outcomes

**Medication**		**total cholesterol**	**clinical events**	**side effects**
	Model	(4) Logistic	(5) Logistic	(6) Logistic
statin prescription	$\hat{\beta}$/OR	-24.82***	13.46***	1.27***
	p-value	(<0.0001)	(<0.0001)	(<0.0001)
	95%CI	[-25.8,-23.9]	[11.1,16.3]	[1.21,1.34]
after 2019	$\hat{\beta}$/OR	7.16***	0.79	1.41***
	p-value	(<0.0001)	(0.1049)	(<0.0001)
	95%CI	[5.74,8.57]	[0.57,1.01]	[1.30,1.52]
after 2019 × statin prescription	$\hat{\beta}$/OR	-7.06***	1.51***	1.03
	p-value	(<0.0001)	(0.0013)	(0.4488)
	95%CI	[-8.42,-5.70]	[1.17,1.93]	[0.95,1.11]
t	$\hat{\beta}$/OR	2.35***	0.90	0.83***
	p-value	(<0.0001)	(0.1831)	(<0.0001)
	95%CI	[1.52,3.19]	[0.78,1.05]	[0.81,0.86]
t^2	$\hat{\beta}$/OR	-0.18***	1.02***	1.02***
	p-value	(<0.0001)	(0.0003)	(<0.0001)
	95%CI	[-0.25,-0.11]	[1.01,1.04]	[1.01,1.02]
t^3	$\hat{\beta}$/OR	0.00*	1.00***	1.00***
	p-value	(0.0145)	(<0.0001)	(<0.0001)
	95%CI	[0.00,0.00]	[1.00,1.00]	[1.00,1.00]
female	$\hat{\beta}$/OR	16.43***	0.29***	1.17***
	p-value	(<0.0001)	(<0.0001)	(<0.0001)
	95%CI	[15.1,17.7]	[0.24,0.34]	[1.12,1.23]
age (reference: >80)				
<50	$\hat{\beta}$/OR	7.50***	0.03***	1.49***
	p-value	(<0.0001)	(<0.0001)	(<0.0001)
	95%CI	[5.70,9.30]	[0.02,0.04]	[1.39,1.60]
50-65	$\hat{\beta}$/OR	12.68***	0.12***	1.29***
	p-value	(<0.0001)	(<0.0001)	(<0.0001)
	95%CI	[11.3,14.1]	[0.09,0.15]	[1.22,1.37]
65-80	$\hat{\beta}$/OR	5.91***	0.36***	1.03
	p-value	(<0.0001)	(<0.0001)	(0.2347)
	95%CI	[4.79,7.03]	[0.30,0.43]	[0.98,1.09]
health service demand of the previous year				
practice visits	$\hat{\beta}$/OR	-0.30***	1.00	1.03***

	p-value	(<0.0001)	(0.5891)	(<0.0001)
	95%CI	[-0.35,-0.24]	[0.99,1.01]	[1.03,1.04]
hospitalization	$\hat{\beta}$/OR	-0.04	1.04***	1.00
	p-value	(0.6413)	(0.0002)	(0.6605)
	95%CI	[-0.20,0.13]	[1.02,1.07]	[0.99,1.01]
specialist visit	$\hat{\beta}$/OR	-0.03	1.00	1.01***
	p-value	(0.4216)	(0.9238)	(<0.0001)
	95%CI	[-0.04,0.10]	[0.99,1.01]	[1.01,1.02]
cardiologist visit	$\hat{\beta}$/OR	-3.81***	2.30**	1.06**
	p-value	(<0.0001)	(<0.0001)	(0.0149)
	95%CI	[-4.67,-2.95]	[2.04,2.60]	[1.01,1.10]
risk factors (measured in the previous year)				
smoking	$\hat{\beta}$/OR	-0.10	0.95	1.13***
	p-value	(0.8768)	(0.6230)	(0.0001)
	95%CI	[-1.39,1.18]	[0.76,1.18]	[1.06,1.21]
hypertension	$\hat{\beta}$/OR	-0.13	0.92	1.14***
	p-value	(0.7322)	(0.1913)	(<0.0001)
	95%CI	[-0.89,0.63]	[0.82,1.04]	[1.10,1.18]
diabetes mellitus	$\hat{\beta}$/OR	-5.71***	1.15	1.03
	p-value	(<0.0001)	(0.0819)	(0.2220)
	95%CI	[-6.74,-4.69]	[0.98,1.34]	[0.98,1.09]
asthma/chronic obstructive pulmonary disease	$\hat{\beta}$/OR	0.48	0.97	1.15***
	p-value	(0.4009)	(0.7025)	(<0.0001)
	95%CI	[-0.63,1.58]	[0.81,1.15]	[1.10,1.21]
obesity	$\hat{\beta}$/OR	-0.83	1.33*	1.16***
	p-value	(0.3164)	(0.0235)	(<0.0001)
	95%CI	[-2.46,0.80]	[1.04,1.69]	[1.08,1.24]
migraine/chronic headache	$\hat{\beta}$/OR	0.08	0.81	1.40***
	p-value	(0.9433)	(0.4213)	(<0.0001)
	95%CI	[-2.21,2.38]	[0.49,1.34]	[1.28,1.54]
	n	52146	159044	159044

OR: Odds Ratio, $\hat{\beta}$: estimated coefficients; individual practice and patient level effects; *p*-values in parentheses; 95% confidence intervals in brackets; significance levels: $^{*}p < 0.05$, $^{**}p < 0.01$, $^{***}p < 0.001$

Table S4. Distribution of Statin prescriptions with different dosages, n (%)

Statin	total	5mg	10mg	20mg	30mg	40mg	60mg	80mg
Simvastatin	20787 (59.4)	59 (0.2)	1828 (5.2)	10275 (29.4)	992 (2.8)	7153 (20.5)	146 (0.4)	334 (1.0)
Lovastatin	192 (0.5)	0 (0)	5 (0.0)	70 (0.2)	0 (0)	117 (0.3)	0 (0)	0 (0)
Pravastatin	1576 (4.5)	0 (0)	123 (0.4)	671 (1.9)	102 (0.3)	680 (1.9)	0 (0)	0 (0)
Fluvastatin	303 (0.9)	0 (0)	0 (0)	94 (0.3)	0 (0)	97 (0.3)	0 (0)	112 (0.3)
Atorvastatin	11439 (32.7)	0 (0)	3190 (9.1)	4742 (13.6)	167 (0.5)	2917 (8.3)	121 (0.3)	302 (0.9)
Rosuvastatin	1674 (4.8)	417 (1.2)	627 (1.8)	446 (1.3)	0 (0)	184 (0.5)	0 (0)	0 (0)
Pitavastatin	0 (0)	0 (0)	0 (0)	0 (0)	0 (0)	0 (0)	0 (0)	0 (0)

n=34973 total patient/year observations with statin prescriptions

Table S5. Distribution lipid-lowering therapy, n (%)

Therapy	ATC[a]	n (%)
statin lipid-lowering therapy		
Simvastatin	C10AA01	20787 (11.1)
Lovastatin	C10AA02	192 (0.1)
Pravastatin	C10AA03	1576 (0.8)
Fluvastatin	C10AA04	303 (0.2)
Atorvastatin	C10AA05	11439 (6.1)
Rosuvastatin	C10AA07	1674 (0.9)
Pitavastatin	C10AA08	0 (0)
statin lipid-lowering combination		
Simvastatin and Ezetimibe	C10BA02	0 (0)
Pravastatin und Fenofibrat	C10BA03	0 (0)
Atorvastatin und Ezetimib	C10BA05	0 (0)
Rosuvastatin und Ezetimib	C10BA06	0 (0)
Atorvastatin und Amlodipin	C10BX03	0 (0)
Rosuvastatin und Acetylsalicylsäure	C10BX05	0 (0)
Atorvastatin, Acetylsalicylsäure und Ramipril	C10BX06	0 (0)
Rosuvastatin und Amlodipin	C10BX09	0 (0)
Atorvastatin, Amlodipin und Perindopril	C10BX11	0 (0)
Atorvastatin, Amlodipin und Ramipril	C10BX18	0 (0)
non-Statin Lipid Lowering Therapies		
Bezafibrat	C10AB02	234 (0.1)
Gemfibrozil	C10AB04	35 (0.0)
Fenofibrat	C10AB05	478 (0.3)
Colestyramin	C10AC01	14 (0.0)
Colesevelam	C10AC04	0 (0)
Ezetimib	C10AX09	0 (0)
Evolocumab	C10AX13	0 (0)
Alirocumab	C10AX14	0 (0)
Inclisiran	C10AX16	0 (0)

n=186450 total patient/year observations with hypecholesterol diagnosis;
[a]Anatomical Therapeutic Chemical code

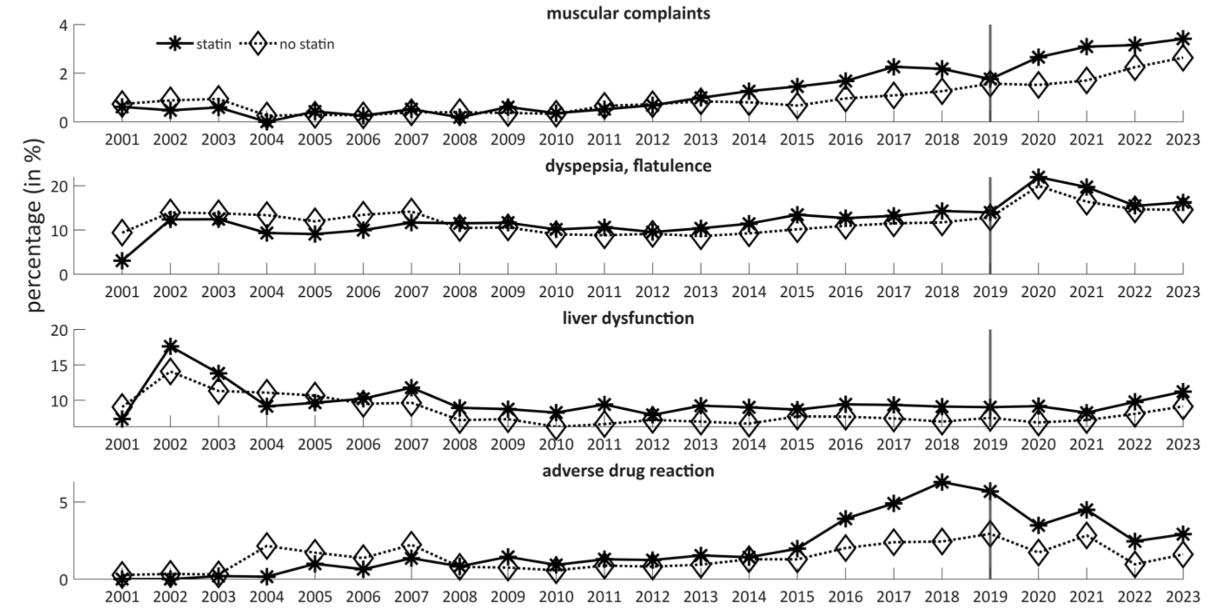

Figure S1. Subcategories of side effects over time

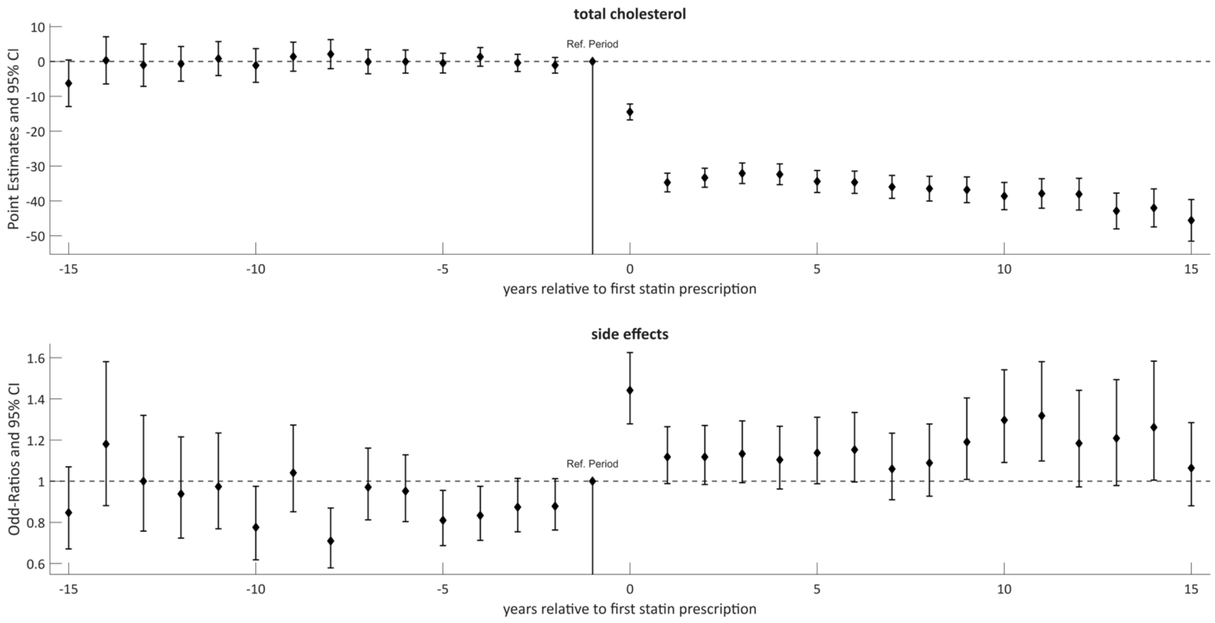

Figure S2. Event time plot

Notes: Point estimates are displayed along with their 95% CIs. The baseline (omitted) base period (Ref. Period) is one year prior to the first statin prescription, indicated by the vertical line. For the total cholesterol, a two-way fixed effects linear model was estimated. For the side effects, a fixed effects conditional logit with year fixed effects was estimated. Estimating an unconditional logit model using dummy variables for each patient produces almost identical results.
